# Study on the influencing factors of college students’ healthy lifestyles based on the capability, opportunity, motivation–behavior model

**DOI:** 10.3389/fpubh.2026.1730314

**Published:** 2026-01-21

**Authors:** Xinyu Liu, Pengyu Lou, Wenjie Teng, Xiaoyan Xu, Zhaofeng Yu, Xiang Li, Liming Lin, Meng Zhao, Zhiwei Dong

**Affiliations:** 1 School of Management, Shandong Second Medical University, Weifang, China; 2 Qingdao Traditional Chinese Medicine Hospital (Qingdao Hiser Hospital Affiliated of Qingdao University), Qingdao, China; 3 School of Media, Qufu Normal University, Rizhao, China; 4 School of Internet Economics and Business, Fujian University of Technology, Fuzhou, China

**Keywords:** college students, COM-B model, healthy lifestyles, mediating effect, social responsibility

## Abstract

**Introduction:**

The college period represents a pivotal stage for establishing long-term health behaviors. Grounded in the COM-B (Capability, Opportunity, Motivation—Behavior) model, this study constructs a comprehensive behavioral explanatory framework: positioning eHealth literacy as Capability, social support as Opportunity, and social responsibility as core Motivation to predict healthy lifestyles (Behavior). It further examines how this mechanism is moderated by major, mental health, and sense of hope.

**Methods:**

From May to June 2022, a cross-sectional survey was conducted involving 4,036 students recruited from six universities in Shandong Province, China, using stratified cluster sampling. Validated scales were administered to assess healthy lifestyle, eHealth literacy, social support, social responsibility, mental health, and sense of hope. Multiple linear regression combined with the Bootstrap method was used to explore variable mediation, and Bootstrap was further applied to test moderation effects.

**Results:**

A total of 4,036 participants were included in this study, among whom 1,811 were male (44.9%) and 2,225 were female (55.1%). In terms of grade distribution, there were 2,216 lower-grade students (55.0%) and 1,820 upper-grade students (45.0%). The mean score for healthy lifestyles was 3.95 (±0.74). Social responsibility emerged as the strongest predictor (*β* = 0.449, 95%CI [0.413, 0.466]) and was found to partially mediate the relationships of both eHealth literacy and social support with healthy lifestyles. Furthermore, major negatively moderated the relationship between eHealth literacy and healthy lifestyles (*β* = −0.127, *p* < 0.001), while mental health status negatively moderated the relationship between social support and healthy lifestyles (*β* = −0.087, *p* < 0.001). In contrast, sense of hope positively moderated the relationship between social responsibility and healthy lifestyles (β = 0.040, *p* < 0.001).

**Discussion:**

Social responsibility is not only related to the healthy lifestyle of college students, but also plays a mediating role between electronic health literacy, social support, and healthy lifestyle. Therefore, colleges and universities should focus on cultivating students’ sense of social responsibility, systematically improve their health literacy, create a supportive campus environment, and enhance students’ psychological capital and sense of hope. These multi-level measures, when working together, will help to establish a sustainable mechanism for promoting a healthy lifestyle among college students.

## 1 Introduction

The “Healthy China 2030” blueprint elevates health to a national strategic priority, explicitly advocating for nationwide healthy lifestyle initiatives (1). Correspondingly, the “Guidelines for Health Education in Regular Higher Education Institutions” underscore the role of universities in cultivating healthy lifestyles to support students’ holistic development (2). The urgency of these policies is starkly highlighted by contemporary challenges. Recent evidence indicates a persistent decline in the physical fitness of Chinese college students, characterized by issues such as rising obesity rates and inadequate cardiorespiratory endurance (3, 4), and 86% of college students have reported health problems in the past year (5). The decline of college students’ physical fitness not only directly weakens the healthy capital of the young labor force, but also poses a systematic threat to the national human capital reserve and public health system through intergenerational accumulation effect in the long run. Therefore, in order to solve this serious public health problem, it is particularly important and urgent to systematically reveal the influencing mechanism and complex causes of healthy lifestyle from the theoretical source, so as to provide scientific basis for formulating accurate intervention strategies.

As a positive behavioral pattern, healthy lifestyles are crucial for disease prevention and the maintenance and enhancement of health (6). A large number of studies have consistently pointed out that the overall lifestyle of current college students urgently needs systematic improvement (7–9). The academic community has conducted extensive exploration of the relevant influencing factors of healthy lifestyles: the existing evidence reveals external situational factors such as gender, grade, and being an only child (10–13). Studies indicate that being female and in a higher grade are protective factors for health literacy, thereby promoting healthier lifestyle practices (11, 12). However, although the existing research has accumulated a wealth of empirical findings, most of the explorations fail to put these scattered influencing factors within a unified theoretical framework and reveal their internal relations and synergistic mechanisms. This current research status limits the overall and systematic understanding of the formation mechanism of college students’ healthy lifestyles.

The Capability-Opportunity-Motivation-Behavior Model (COM-B), as the core part of the behavior change wheel, was proposed by Michie et al. (14). The model posits that the enactment and change of behavior rely on the synergistic interaction of capability, opportunity and motivation, thereby providing a comprehensive and systematic framework for understanding the determinants of individual health behavior. Many studies (15–17) also show that the COM-B model has been widely used in disease prevention, self-management and behavior change in health promotion. Therefore, with the COM-B model as its theoretical framework, this study seeks to systematically identify and examine the key capability, opportunity, and motivation factors associated with healthy lifestyles of Chinese college students, and to develop an integrated theoretical model for the formation mechanism of their healthy behaviors.

## 2 Literature review and hypothesis formulation

### 2.1 Literature review

Within the COM-B model, capability includes physical/psychological status and knowledge/skills (18), serving as the key to behavioral execution. College students are the main group accessing online health information, which is a key factor associated with their healthy lifestyles. eHealth literacy—defined as the core capability to evaluate and apply such information—is directly correlated with the scientific validity and sustainability of their healthy lifestyles (18). The operating mechanism of this capability can be explained by the information processing process revealed by the Bayesian Mind sponge Framework (BMF) (19). This framework points out that when individuals encounter online health information, they evaluate it against their existing health-related cognitive-behavioral schemata, subsequently engaging in selective processing: information consistent with scientific evidence is “absorbed” and integrated, whereas conflicting or unsubstantiated information is “filtered out” (19). The level of eHealth literacy determines the efficiency of this information processing. Individuals with high eHealth literacy can establish scientific cognitive benchmarks, accurately identify valuable content during information screening, and translate it into health behaviors, with research showing significantly higher correct health behavior rates among this group (20). Therefore, as the core manifestation of the capability dimension in the COM-B model, the role of eHealth literacy is realized precisely through the mind sponge mechanism of the BMF. Together, they construct a comprehensive capability system that bridges cognitive processing and behavioral transformation.

Opportunity includes the environmental and social factors that facilitate a behavior (21). Social support, as a key opportunity factor, systematically creates favorable conditions for college students to practice healthy behaviors through three levels: interpersonal networks, learning and living environments, and information environments (22). At the interpersonal network level, emotional support from family members, friends and teachers is the foundation. The care from family members and the encouragement from peers can enhance students’ psychological motivation to adhere to healthy behaviors (23, 24). At the level of the learning and living environment, as the main venue for daily activities, the institutional and organizational support provided by universities is of vital importance. A supportive campus environment can significantly enhance students’ willingness and possibility to adopt a healthy lifestyle. Studies have shown that a well-planned campus-built environment is conducive to college students’ physical exercise behaviors (25). At the information environment level, health information platform services help students form scientific cognition and promote the generation of healthy behaviors (26).

Motivation involves the brain processes that energize and direct behavior, encompassing emotional responses, habitual patterns, and analytical decision-making (21). To gain a deeper understanding of the formation mechanism of motivation, this study introduces the value theory and draws on the Granular Interaction Thinking Theory (GITT). Value theory (27) holds that the formation of value is an information processing process aimed at reducing psychological uncertainty. This process depends on GITT, which is developed with the psychological mechanism described by MT as its most fundamental conception and starting point, and MT is also the core mechanism on which BMF relies (28). GITT believes that individuals transform various influencing factors into different attributes of “information granularity” in behavioral decision-making (29). Through the dynamic interaction of multi-granularity information, they re-evaluate and integrate their own values, ultimately forming stable and behavior-oriented motivations. Social responsibility, including individual health responsibility and public health responsibility, plays an important role in promoting healthy lifestyle. Individual health responsibility is the basis and premise of healthy behavior (30), while public health responsibility contributes to the formation of individual and collective health behavior and the formation of healthy lifestyle (31). This motivation perspective based on value theory reveals the dynamics and situational dependence of motivation formation, providing a new theoretical perspective for understanding the persistence mechanism of healthy behaviors.

In the above theoretical framework, in addition to the core components, factors including students’ academic major, mental health, and sense of hope also play a key moderating role, and are closely correlated with the transformation efficiency among capability, opportunity, and motivation. Academic major functions as a moderator in the relationship between capability and behavior. The knowledge structure and thinking mode shaped by students’ academic majors form a cognitive framework that is directly linked to the efficiency of translating eHealth literacy into practical behaviors (32). Mental health status regulates the relationship between opportunity and behavior. Mental health levels can promote the formation of self-efficacy (33), enabling individuals to independently cope with various challenges. Even in the absence of social support, they can maintain a healthy lifestyle by leveraging their psychological advantages (34). Sense of hope is an important moderating variable between motivation and behavior. As a positive psychological trait facing the future, high hope can enhance individuals’ confidence and motivation belief in the path to achieve health goals, thus significantly strengthening the driving effect of reflective motivation on health behavior (35). When individuals have firm expectations for future health outcomes, their willingness and perseverance to adhere to a healthy lifestyle will be significantly improved.

**Figure 1:**
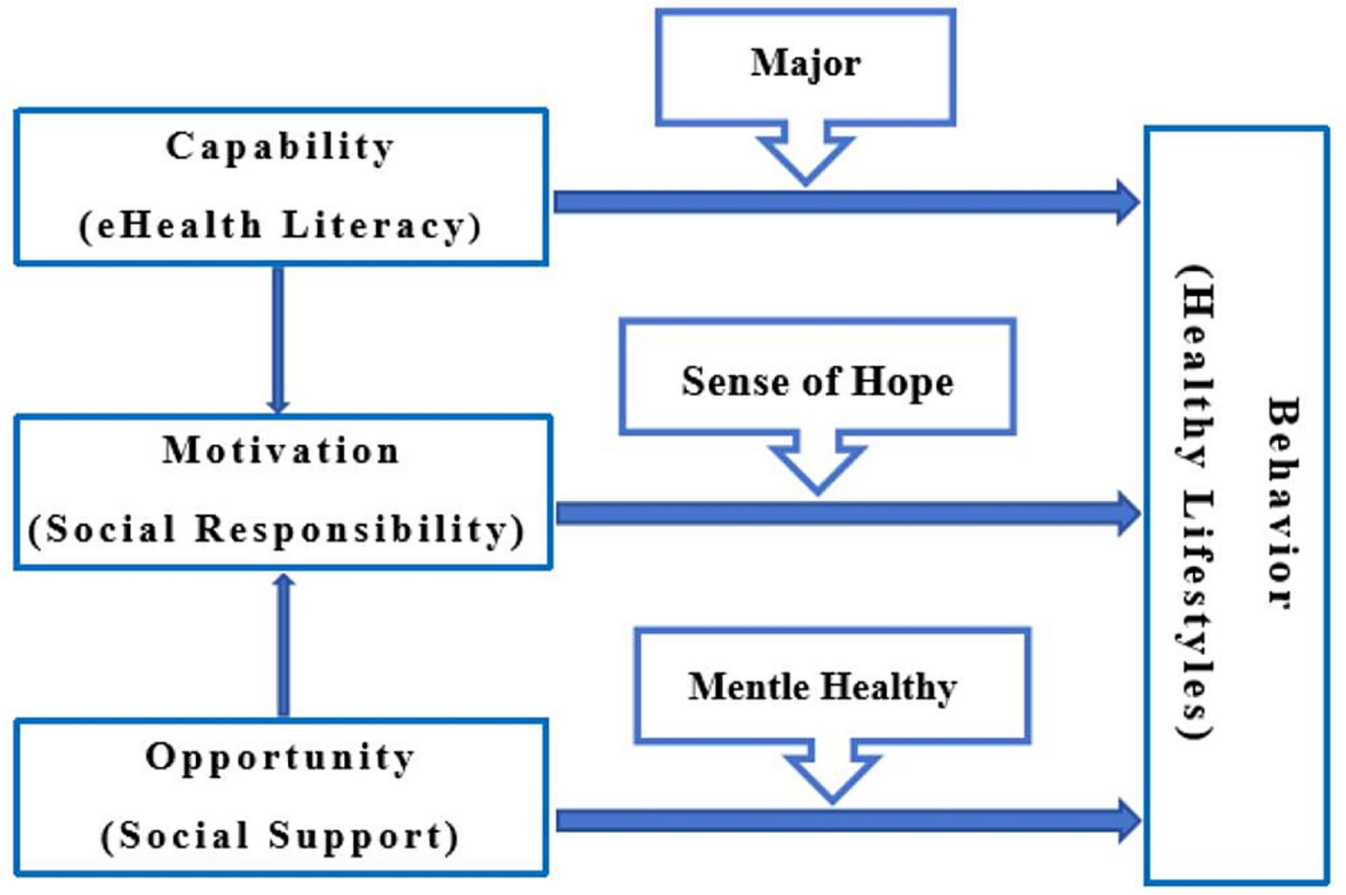
Theoretical model of college students’ healthy lifestyle based on COM-B.

Therefore, this study adopts the COM-B model as the core framework and integrates BMF and value theory for theoretical enhancement, constructing a comprehensive model of the formation mechanism of healthy lifestyles among college students (Figure 1). This model not only systematically examines the driving effects of eHealth literacy (capability), social support (opportunity), and social responsibility (motivation) on college students’ healthy lifestyles, but also reveals the moderating mechanisms of major, sense of hope, and mental health in the above pathways. This research is expected to provide an integrated theoretical perspective for understanding the complex causes of college students’ health behaviors and offer empirical evidence for developing precise health promotion intervention strategies.

### 2.2 Hypothesis formulation

#### 2.2.1 Positive relationship between social responsibility and healthy lifestyles

Although most college students have an average or high level of electronic health literacy, and can effectively obtain, evaluate and use online health information (36), and universities themselves provide social support for students’ health by offering health knowledge courses and promoting health literacy (37), survey data from many countries show that the health status of college students is relatively poor (3–5). This indicates that compared with ability and opportunity, motivation may be the most important factor in promoting a healthy lifestyle among college students.

Social responsibility serves as a core intrinsic motivation driving the adoption of healthy lifestyles among college students, manifested through the concrete practice of health responsibility and public health responsibility (30, 31). Health responsibility is the internalization process of an individual’s awareness and commitment to their own health behaviors (38), while public health responsibility refers to fulfilling the social obligation of reducing group health risks by practicing preventive health behaviors. When individuals have a strong sense of social responsibility, they tend to actively choose a healthy lifestyle to maintain their own and others’ health. Previous studies have confirmed that a sense of social responsibility can effectively promote the generation and practice of healthy behaviors among individuals (39). Based on this logical chain, this study proposes the core hypothesis:

*H1:* Social responsibility is positively associated with college students’ healthy lifestyles.

#### 2.2.2 Mediating role of social responsibility in the relationship between eHealth literacy and healthy lifestyles

Enhanced eHealth literacy strengthens an individual’s ability to process online health information, thereby fostering the formation and development of a sense of social responsibility (40). Individuals with high literacy levels are more proactive in utilizing digital health information. This not only increases their initiative in personal health management but also enables them to disseminate knowledge via social media, cultivating a dual sense of responsibility towards both personal health and public health issues (41). Studies have found that college students with high eHealth literacy can focus on and judge pandemic information more rationally (42), demonstrating their awareness of fulfilling public health responsibilities through digital channels. This actively promotes taking on social health obligations, achieving the integration of personal health and social responsibility. Based on this logic, the study proposes the following hypothesis:

*H2a:* Social responsibility mediates the relationship between eHealth literacy and healthy lifestyles among college students.

Social support serves as a critical foundation for college students to fulfill their social responsibility, and establishing an extensive network of social support is highly significant for strengthening their sense of social responsibility (43). Family support can alleviate external pressures, generate positive health outcomes, and is significantly associated with higher levels of students’ social responsibility (44). Furthermore, positive interpersonal relationships are positively linked to individuals’ sense of social responsibility, whereby better interpersonal relationships correlate with greater engagement in responsible behaviors (45). Research targeting college students with left-behind experiences indicates a significant positive correlation between their perceived social support and responsible cognition, emotion, and behavior—the greater the support, the stronger the sense of responsibility (46). Richer social support more effectively motivates students to participate in collective and social affairs. Based on this, the study proposes the following hypothesis:

*H2b:* Social responsibility mediates the relationship between social support and healthy lifestyles among college students.

#### 2.2.3 Moderating effects of major, mental health, and sense of hope on healthy lifestyles

Major, as a key variable reflecting disparities in college students’ health knowledge reserves and cognitive patterns, correlates with the development of their health cognition systems and information appraisal competencies (32). It critically differentiates the foundation for forming healthy lifestyles, the efficiency of translating knowledge into behavior, and the priority of needs regarding eHealth literacy. Students in medical majors, due to systematic medical education and clinical exposure, have established rigorous health knowledge systems, where improvements in eHealth literacy offer limited additional benefits to their healthy lifestyles (47). In contrast, non-medical majors often possess fragmented health knowledge, and enhancing their eHealth literacy can effectively bridge this gap and optimize their healthy lifestyles (32). Based on this, the study proposes the following hypotheses:

*H3a:* Major moderates the relationship between eHealth literacy and healthy lifestyles.

*H3b:* Compared to students in medical majors, eHealth literacy has a stronger positive predictive effect on the healthy lifestyle of non-medical major students.

Mental health refers to the state in which an individual’s mind develops to an optimal level without conflicting with the mental well-being of others in terms of physical, intellectual, and emotional aspects (33). Students with lower levels of mental health are more susceptible to developing negative emotions such as anxiety and depression when confronted with life stressors and emotional distress, often lacking effective coping strategies (48). For these individuals, social support serves as a crucial external resource, providing emotional comfort, practical assistance, and constructive guidance, which significantly aids in the adoption of healthy lifestyles (49, 50). Conversely, students with higher levels of mental health possess stronger psychological adjustment and self-management capabilities. They are better equipped to handle challenges independently and are less reliant on external social support. Even in the absence of strong support systems, they can maintain healthy lifestyles by leveraging their intrinsic psychological strengths. Based on this analysis, the study proposes the following hypotheses:

*H4a:* Mental health moderates the relationship between social support and healthy lifestyles.

*H4b:* Compared to college students with higher levels of mental health, social support has a stronger positive predictive effect on the healthy lifestyle of college students with lower levels of mental health.

Sense of hope refers to an individual’s positive belief in their ability to discover pathways to achieve goals and to continuously generate and sustain the motivation required to pursue them (51). College students with higher levels of hope can effectively mobilize their internal positive resources when facing complex challenges. In such circumstances, social responsibility and sense of hope work synergistically to further strengthen their goal commitment and drive for action. When these students integrate social responsibility with their personal development goals, they become more conscious of transforming it into systematic self-improvement plans. Since healthy lifestyles form a crucial foundation for achieving personal goals and making societal contributions, they proactively incorporate such practices into their daily routines (52). Based on this analysis, the study proposes the following hypotheses:

*H5a:* Sense of hope moderates the relationship between social responsibility and healthy lifestyles.

*H5b:* Compared to college students with lower levels of sense of hope, social responsibility has a stronger positive predictive effect on the healthy lifestyle of college students with higher levels of sense of hope.

## 3 Materials and methods

### 3.1 Study design

A cross-sectional survey design was adopted. Methodologically, this study aimed to be an observational study that describes the current state of healthy lifestyles among college students and, more importantly, tests the specific mediating and moderating mechanisms proposed within the COM-B framework.

### 3.2 Participants

The participants were 4,036 undergraduate students from 6 universities in Shandong Province. The inclusion criteria were: (1) full-time undergraduate students (2) voluntary participation. Exclusion criteria comprised individuals with serious health conditions that prevented engagement in normal physical exercise and other basic daily activities.

### 3.3 Sample size determination and sampling procedure

From May to June 2022, by using the stratified cluster sampling method, six universities were selected in Shandong Province based on the nature of their disciplines: 2 comprehensive universities (Qingdao University and Ludong University), 2 science and engineering universities (Qingdao University of Technology and Weifang Institute of Technology), and 2 medical universities (Weifang Medical University and Binzhou Medical University). First, randomly select four majors from each university; Secondly, students are stratified by grade. One class is randomly selected from each grade, and a questionnaire survey is conducted among all the undergraduate students in the selected class (with a total of 127,985 students from 6 universities, and a sampling ratio of 3% is adopted (53), the overall sample size should be no less than 3,840 people). A total of 4,320 questionnaires were distributed, among which 4,036 were valid, with an effective rate of 93.4%.

### 3.4 Instruments and tools

#### 3.4.1 Dependent variable

Healthy lifestyles were evaluated using the evaluation scale revised by Jiao et al. (54), which contains 33 items and covers 8 dimensions: exercise behavior (3items), regular living behavior (3items), dietary and nutritional behavior (4items), health hazard behavior (2items), health responsibility behavior (5items), interpersonal relationship behavior (6items), appreciation of life behavior (5items), stress management behavior (5items). This scale was scored as a 5-point frequency scale: (1 = “never,” 2 = “seldom,” 3 = “sometimes,” 4 = “often,” 5 = “always,”) with higher scores indicating more frequent engagement in healthy lifestyle behaviors. In this study, the Cronbach’s *α* value of the scale is 0.721, and its composite reliability is 0.717, indicating that the reliability of the scale is good.

#### 3.4.2 eHealth literacy

The measurement of eHealth literacy adopted the Chinese version of the eHEALS scale, which was originally developed by Norman et al. (55) and revised by Guo et al. Chinese version of the eHEALS (56). This 8-item scale comprises three dimensions, assessing individuals’ capacity to access, evaluate, and apply online health information. This scale adopts the Likert 5-point scoring method (1 = “strongly disagree,” 2 = “disagree,” 3 = “neutral,” 4 = “agree,” 5 = “strongly agree”) where higher scores reflect a better eHealth literacy. In this study, the Cronbach’s *α* value of the scale is 0.714, and its composite reliability is 0.740, indicating that the reliability of the scale is good.

#### 3.4.3 Social support

Social support was assessed using a scale adapted from the University Student Health Ecological Environment Assessment Tool (57). This 12-item instrument measures three dimensions: the interpersonal network layer (family relationships, teacher-student relationships, and peer interactions), the learning and living environment layer (health education curriculum content, teaching methods, health practices, sports facilities, dining quality, and accommodation conditions), and the information environment layer (health knowledge promotion, health information platform services, and health needs communication). This scale adopts the Likert 5-point scoring method (1 = “strongly disagree,” 2 = “disagree,” 3 = “neutral,” 4 = “agree,” 5 = “strongly agree”) where higher scores reflect a stronger social support. In this study, the Cronbach’s *α* value of the scale is 0.7, and its composite reliability is 0.726, indicating that the reliability of the scale is good.

#### 3.4.4 Social responsibility

Social responsibility was assessed using the University Student Social Responsibility Cognition and Behavior Development Scale (58). This 40-item instrument comprises three dimensions: cognition, identification, and the developmental stage of social responsibility behavior. This scale adopts the Likert 5-point scoring method (1 = “very inconsistent,” 2 = “inconsistent,” 3 = “uncertain,” 4 = “consistent,” 5 = “very consistent”) where higher scores reflect a stronger social responsibility. In this study, the Cronbach’s *α* value of the scale is 0.798, and its composite reliability is 0.816, indicating that the reliability of the scale is good.

#### 3.4.5 Moderating variables

This study selected major, mental health, and sense of hope as moderating variables.

Major was assessed using a single item: “What is your major type?” Responses were dichotomized as “non-medical major” (coded as 0) and “medical major” (coded as 1).

Mental health was evaluated using a 27-item scale encompassing six dimensions: happiness experience, interpersonal harmony, positive learning, emotional regulation, goal pursuit, and challenge acceptance (59). This scale adopts the Likert 5-point scoring method (1 = “very inconsistent,” 2 = “inconsistent,” 3 = “uncertain,” 4 = “consistent,” 5 = “very consistent”) where higher scores reflect a better mental health. In this study, the Cronbach’s *α* value of the scale is 0.819, and its composite reliability is 0.803, indicating that the reliability of the scale is good.

The sense of hope was measured using the Hope Trait Scale developed by Snyder et al. (51) and revised by Chen et al. (60). This revised Chinese version contains a total of 12 items, covering two core dimensions: pathway thinking (4 items) and agency thinking (4 items). The remaining four are fill-in items designed to divert the subjects’ attention and are not included in the total score calculation. Responses were collected on a 4-point Likert scale (1 to 4), where higher scores reflect a stronger sense of hope. In this study, the Cronbach’s *α* value of the scale is 0.727, and its composite reliability is 0.779, indicating that the reliability of the scale is good.

To examine the structural validity of the scales, confirmatory factor analysis (CFA) was conducted for each variable in this study. The results showed that the Comparative Fit Index (CFI) values for each scale were as follows: Healthy Lifestyles Scale (0.907), eHealth Literacy Scale (0.981), Social Support Scale (0.952), Social Responsibility Scale (0.948), Mental Health Scale (0.865), and Sense of Hope Scale (0.979). With the exception of the Mental Health Scale (CFI = 0.865), which was slightly below the ideal standard of 0.90, the CFI values of all other scales reached excellent levels (> 0.90). Although the CFI value for Mental Health did not meet the ideal threshold, it remained above the acceptable cutoff of 0.85.

### 3.5 Data collection procedure

The survey was conducted anonymously through an online platform (wenjuanxing), with each IP address restricted to a single submission. Additionally, questionnaires with missing basic respondent information, a response time of less than 120 s, or an item missing rate of ≥5% were excluded (61).

### 3.6 Statistical analysis

Data processing and analysis were performed using SPSS version 27.0. The normality test of the data showed that the eHealth literacy, social support, social responsibility, mental health, sense of hope and healthy lifestyles failed to pass the normality test, but the overall distribution was relatively symmetrical, the degree of deviation from the normal distribution was relatively small, and the sample size in this study was large (62, 63). Therefore, in this study, the data were analyzed as if they were in accordance with normal distribution. Continuous variables conforming to normal distribution were described by Mean ± SD, and categorical variables were described by *N* (%). Spearman’s correlations were used to evaluate the associations between social support, social responsibility, eHealth literacy, mental health, sense of hope and college students’ healthy lifestyles, with the correlation coefficient denoted by the symbol *ρ* (rho). We utilized the SPSS PROCESS macro developed by Hayes (64) to examine the mediating role of social responsibility in two pathways: (1) between eHealth literacy and healthy lifestyle, and (2) between social support and healthy lifestyle. For this mediation analysis, we employed the bootstrap method with 5,000 repeated samplings and calculated 95% confidence intervals. Results were deemed statistically significant if the 95% CI did not include zero. Subsequently, we used the same SPSS PROCESS macro (64) to assess the moderating effects of major, mental health, and hope. A statistically significant regression coefficient for the interaction term (*p* < 0.05) was taken as evidence of a moderation effect.

### 3.7 Ethical considerations

This study was approved by the Medical Research Ethics Committee of Shandong Second Medical University (IRB approval no. 2023YX116). Informed consent has been obtained from all subjects and their legal guardians. This study adhered to the Declaration of Helsinki to this effect.

## 4 Results

### 4.1 Basic information of participants

Table 1 provides the basic information about participants. Among the 4,036 respondents included in this study, 1,811 (44.9%) were male and 2,225 (55.1%) were female. There were 2,216 (55.0%) lower-grade students and 1,820 (45.0%) upper-grade students. In terms of major, 991 (24.6%) were medical majors and 3,045 (75.4%) were non-medical majors. Additionally, 1,312 (32.5%) participants were single-child, and 2,724 (67.5%) were not. The average scores of eHealth literacy, social support, social responsibility mental health, sense of hope and healthy lifestyles were 4.25 ± 0.83, 3.92 ± 0.76, 4.37 ± 0.76, 3.36 ± 0.76, 4.25 ± 0.74, 3.95 ± 0.74, respectively. Female, lower-grade, non-medical major, and only-child students had higher healthy lifestyle scores. To clarify the distribution characteristics of the sample across core research variables, this study adopted the equidistant trisection method with balanced adjustment to divide the total scores of the six scales into three levels (low, medium, and high), and calculated their frequencies and percentages (see Table 2). The proportion of participants with a high level of Healthy Lifestyles reached 71.2%, indicating that the majority of respondents had a relatively high level of healthy lifestyles.

**Table 1:** Demographic characteristics of study participants (*N* = 4,036).

Demographic characteristics	*N* (%)	eHealth literacy Mean ± SD	Social support Mean ± SD	Social responsibility Mean ± SD	Mental health Mean ± SD	Sense of hope Mean ± SD	Healthy lifestyles Mean ± SD
Gender							
Male	1811 (44.9)	4.28 ± 0.85	3.87 ± 0.82	4.27 ± 0.83	3.25 ± 0.84	3.21 ± 0.75	3.90 ± 0.81
Female	2,225 (55.1)	4.23 ± 0.81	3.97 ± 0.70	4.45 ± 0.68	3.44 ± 0.77	3.13 ± 0.68	3.98 ± 0.69
F-value		2.737	16.293^***^	59.188^***^	53.572^***^	11.671^***^	49.103^***^
Grade							
Lower-grade	2,216 (55.0)	4.30 ± 0.83	3.96 ± 0.77	4.39 ± 0.77	3.36 ± 0.82	3.18 ± 0.73	3.97 ± 0.76
Upper-grade	1820 (45.0)	4.20 ± 0.82	3.87 ± 0.75	4.35 ± 0.75	3.34 ± 0.77	3.13 ± 0.69	3.91 ± 0.72
F-value		4.786^**^	7.052^***^	3.417^*^	1.312	2.509	4.362^**^
Major							
Non-medical major	3,045 (75.4)	4.26 ± 0.84	3.93 ± 0.79	4.37 ± 0.77	3.32 ± 0.80	3.17 ± 0.71	3.95 ± 0.76
Medical major	991 (24.6)	4.23 ± 0.79	3.89 ± 0.67	4.38 ± 0.72	3.48 ± 0.79	3.10 ± 0.68	3.92 ± 0.70
F-value		1.133	1.714	0.358	29.440^***^	9.930^**^	2.067
Single-child							
Yes	1,312 (32.5)	4.34 ± 0.81	3.96 ± 0.78	4.36 ± 0.81	3.31 ± 0.86	3.24 ± 0.72	3.99 ± 0.79
No	2,724 (67.5)	4.21 ± 0.83	3.91 ± 0.75	4.37 ± 0.76	3.38 ± 0.78	3.13 ± 0.71	3.93 ± 0.72
F-value		20.304^***^	3.866^*^	0.353	6.197^*^	20.733^***^	6.015^*^
Total	4,036 (100)	4.25 ± 0.83	3.92 ± 0.76	4.37 ± 0.76	3.36 ± 0.76	3.16 ± 0.71	3.95 ± 0.74

*p < 0.05, **p < 0.01, *p < 0.001.

**Table 2:** Frequency distribution of variables relevant to healthy lifestyles after equidistant trisection (*N* = 4,036).

Variables	Low level	Medium level	High level
eHealth literacy	77 (1.9)	820 (20.3)	3,139 (77.8)
Social support	45 (1.1)	1,354 (33.5)	2,637 (65.3)
Social responsibility	66 (1.6)	838(20.8)	3,132 (77.6)
Mental health	447 (11.1)	2086 (51.7)	1,503 (37.2)
Sense of hope	108 (2.7)	1,130 (28.0)	2,798 (69.3)
Healthy lifestyles	36(0.9)	1,127(27.9)	2,873(71.2)

### 4.2 Correlation between variables

The results of the correlation analysis are shown in Table 3. Spearman correlation analysis showed that eHealth literacy, social support, social responsibility was all positively correlated with healthy lifestyles (*ρ* = 0.417, *p* < 0.01; ρ = 0.709, *p* < 0.01; ρ = 0.755, *p* < 0.01, respectively). A positive correlation was also observed between eHealth literacy and social responsibility (ρ = 0.431, *p* < 0.01). Furthermore, social support showed a significant positive correlation with social responsibility (ρ = 0.754, *p* < 0.01).

**Table 3:** Correlation (ρ) analysis results (*N* = 4,036).

Variables	Healthy lifestyles	eHealth literacy	Social support	Social responsibility	Mental health	Sense of hope
Healthy lifestyles	1					
eHealth literacy	0.417^**^	1				
Social support	0.709^**^	0.551^**^	1			
Social responsibility	0.755^**^	0.431^**^	0.754^**^	1		
Mental health	0.087^**^	0.130^**^	0.159^**^	0.152^**^	1	
Sense of hope	0.441^**^	0.707^**^	0.551^**^	0.405^**^	0.149^**^	1

**p < 0.01.

### 4.3 Multiple linear regression analysis of factors influencing healthy lifestyles

Covariance diagnostics for the four variables (eHealth literacy, social support, social responsibility, and healthy lifestyles) indicated that the inflation factors were all below 10 (VIF: 1.390–2.367), and the tolerances ranged from greater than 0.1 to less than1 (Tol: 0.423–0.720), confirming the Absence of serious multicollinearity among the variables (see Table 4).

**Table 4:** Multiple linear regression analysis of factors influencing healthy lifestyles.

Variable	β	SE	t	95%CI
eHealth literacy	0.032	0.010	2.785^*^	0.009 ~ 0.049
Social support	0.380	0.015	25.109^**^	0.343 ~ 0.401
Social responsibility	0.449	0.014	32.497^**^	0.413 ~ 0.466

*p < 0.05, **p < 0.01 indicate levels of statistical significance.

The multiple linear regression analysis revealed that eHealth literacy (*β* = 0.032, *p* < 0.01), social support (β = 0.380, *p* < 0.001), and social responsibility (β = 0.449, *p* < 0.001) were all significant positive predictors of healthy lifestyles. Among these, social responsibility demonstrated the highest standardized regression coefficient (β = 0.449).

### 4.4 Mediating role of social responsibility

To elucidate the underlying relationships among social responsibility, eHealth literacy, social support, and healthy lifestyles, two separate mediation analyses were conducted, controlling for demographic characteristics including gender, grade, and single-child status. The first model examined social responsibility as a mediator between eHealth literacy (independent variable) and healthy lifestyles (dependent variable). The second model tested social responsibility as a mediator between social support (independent variable) and healthy lifestyles (dependent variable). The mediation analysis was conducted using the PROCESS macro for SPSS.

**Figure 2:**
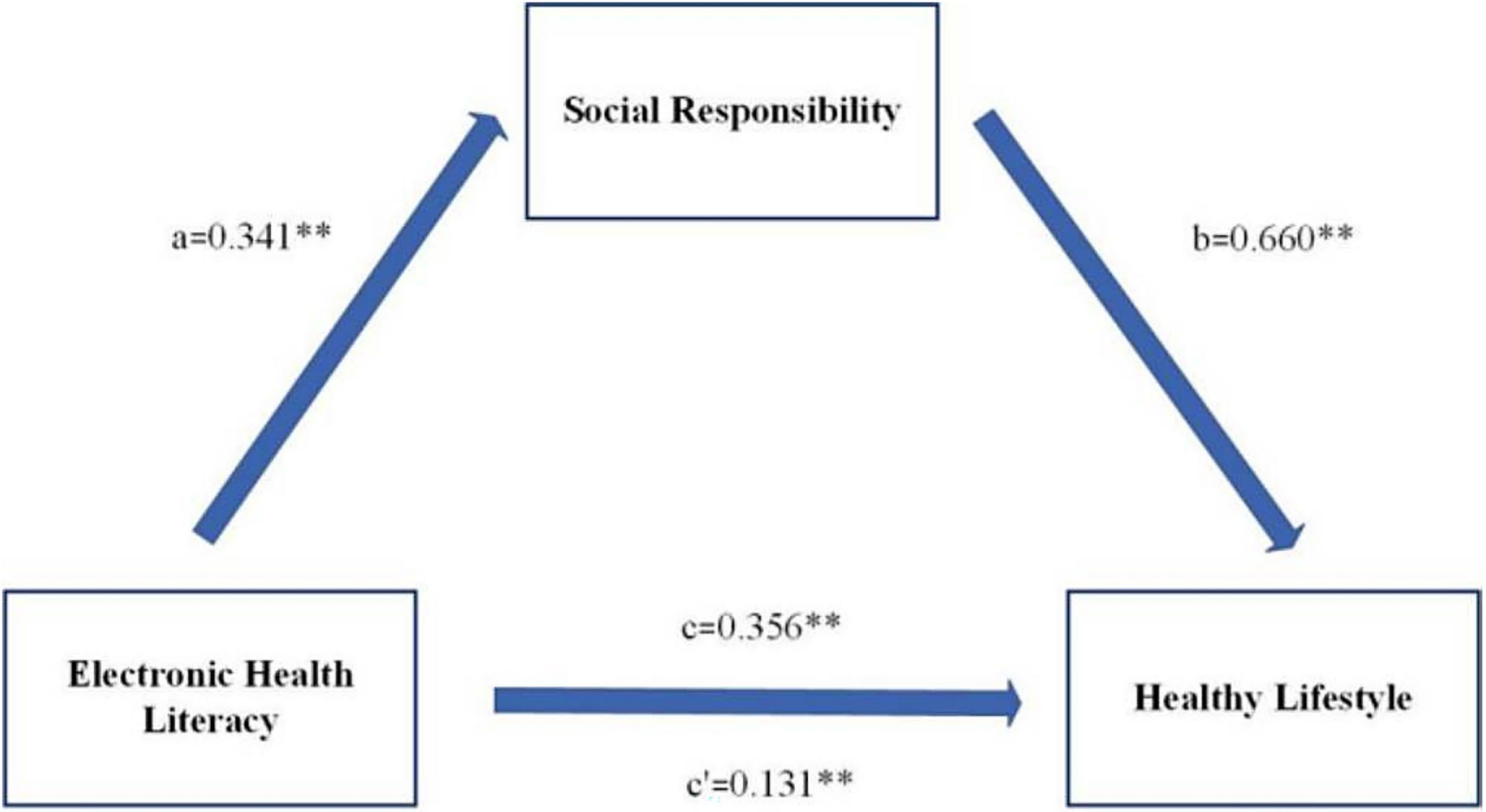
Mediation model of social responsibility between eHealth literacy and healthy lifestyles.

The mediating model of social responsibility between eHealth literacy and healthy lifestyle shows that the total effect of eHealth literacy on healthy lifestyle is 0.356 (*p* < 0.01), and the 95% confidence interval is (0.331, 0.382). eHealth literacy can positively predict social responsibility, with a path coefficient of 0.340 (*p* < 0.01). Social responsibility can positively predict a healthy lifestyle, with a path coefficient of 0.660 (*p* < 0.01). The indirect effect of eHealth literacy on a healthy lifestyle is 0.225, with a 95% confidence interval of (0.202, 0.247), indicating that the mediating effect is valid. After adding the mediating variable (social responsibility), the direct effect of eHealth literacy on a healthy lifestyle was 0.131 (*p* < 0.01), with a 95% confidence interval of (0.111, 0.151), indicating that social responsibility plays a partial mediating role in the relationship between eHealth literacy and a healthy lifestyle. Please refer to Tables 5, 6 and Figure 2 for details.

**Table 5:** Mediating effect of social responsibility.

Model	Result variable	Predictor variable	R^2^	F	Coeff	SE	95%CI	t
Model 1	Social responsibility	Constant	0.152	121.020				
		eHealth literacy			0.340	0.013	0.314 ~ 0.367	25.463**
	Healthy lifestyles	Constant	0.550	705.233				
		eHealth literacy			0.131	0.010	0.111 ~ 0.158	12.799**
		Social responsibility			0.660	0.011	0.638 ~ 0.682	58.817**
Model 2	Social responsibility	Constant	0.492	666.640				
		Social support			0.697	0.011	0.6752 ~ 0.7192	62.198**
	Healthy lifestyles	Constant	0.610	901.691				
		Social support			0.384	0.013	0.3580 ~ 0.4111	28.405**
		Social responsibility			0.444	0.013	0.4173 ~ 0.4706	32.670**

**p < 0.01 indicate levels of statistical significance.

**Table 6:** Bootstrap results for the mediation effects.

Effect type	Effect value	SE	LLCI	ULCI
Direct effect				
eHealth literacy→ Healthy lifestyles	0.131	0.010	0.111	0.151
Social support → Healthy lifestyles	0.384	0.013	0.358	0.411
Indirect effect				
eHealth literacy → Social responsibility → Healthy lifestyles	0.225	0.011	0.202	0.247
Social support → Social responsibility → Healthy lifestyles	0.309	0.014	0.281	0.337
Total effect				
eHealth literacy → Healthy lifestyles	0.356	0.013	0.331	0.382
Social support → Healthy lifestyles	0.694	0.010	0.672	0.715

**Figure 3:**
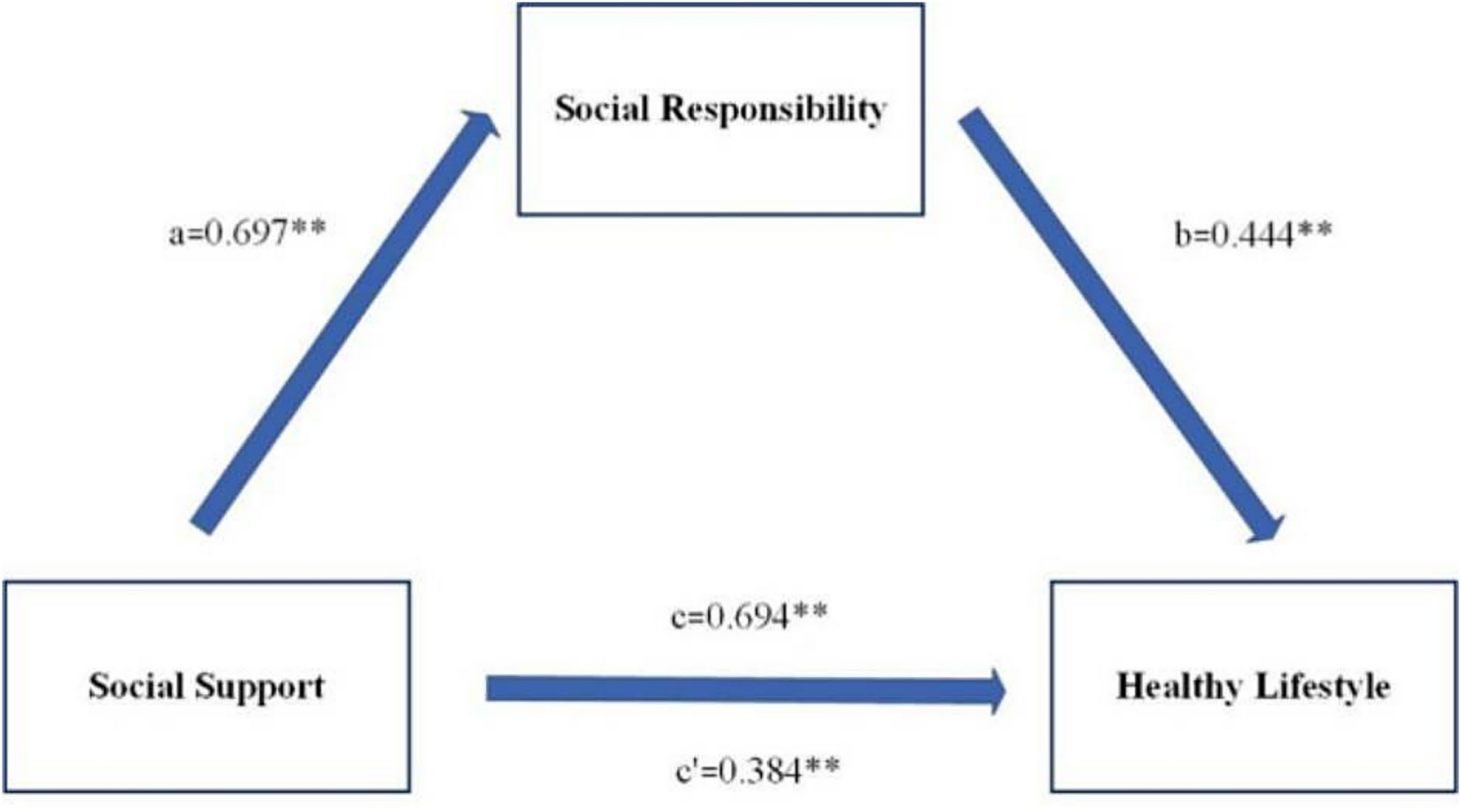
Mediation model of social responsibility between social support and healthy lifestyles.

The mediation model of social responsibility between social support and a healthy lifestyle shows that the total effect of social support on a healthy lifestyle is 0.694 (*p* < 0.01), and the 95% confidence interval is (0.672, 0.715). Social support can positively predict social responsibility, with a path coefficient of 0.697 (*p* < 0.01). Social responsibility can positively predict a healthy lifestyle, with a path coefficient of 0.444 (*p* < 0.01). The indirect effect of social support on a healthy lifestyle is 0.309, with a 95% confidence interval of (0.281, 0.337), indicating that the mediating effect holds. After adding the mediating variable (social responsibility), the direct effect of social support on a healthy lifestyle was 0.384 (*p* < 0.01), with a 95% confidence interval of (0.358, 0.411), indicating that social responsibility plays a partial mediating role in the relationship between social support and a healthy lifestyle. Please refer to Tables 5, 6 and Figure 3 for details.

### 4.5 Moderation effect analysis

The moderation analysis was conducted using the PROCESS macro for SPSS, controlling for gender, grade, and single-child status.

**Figure 4:**
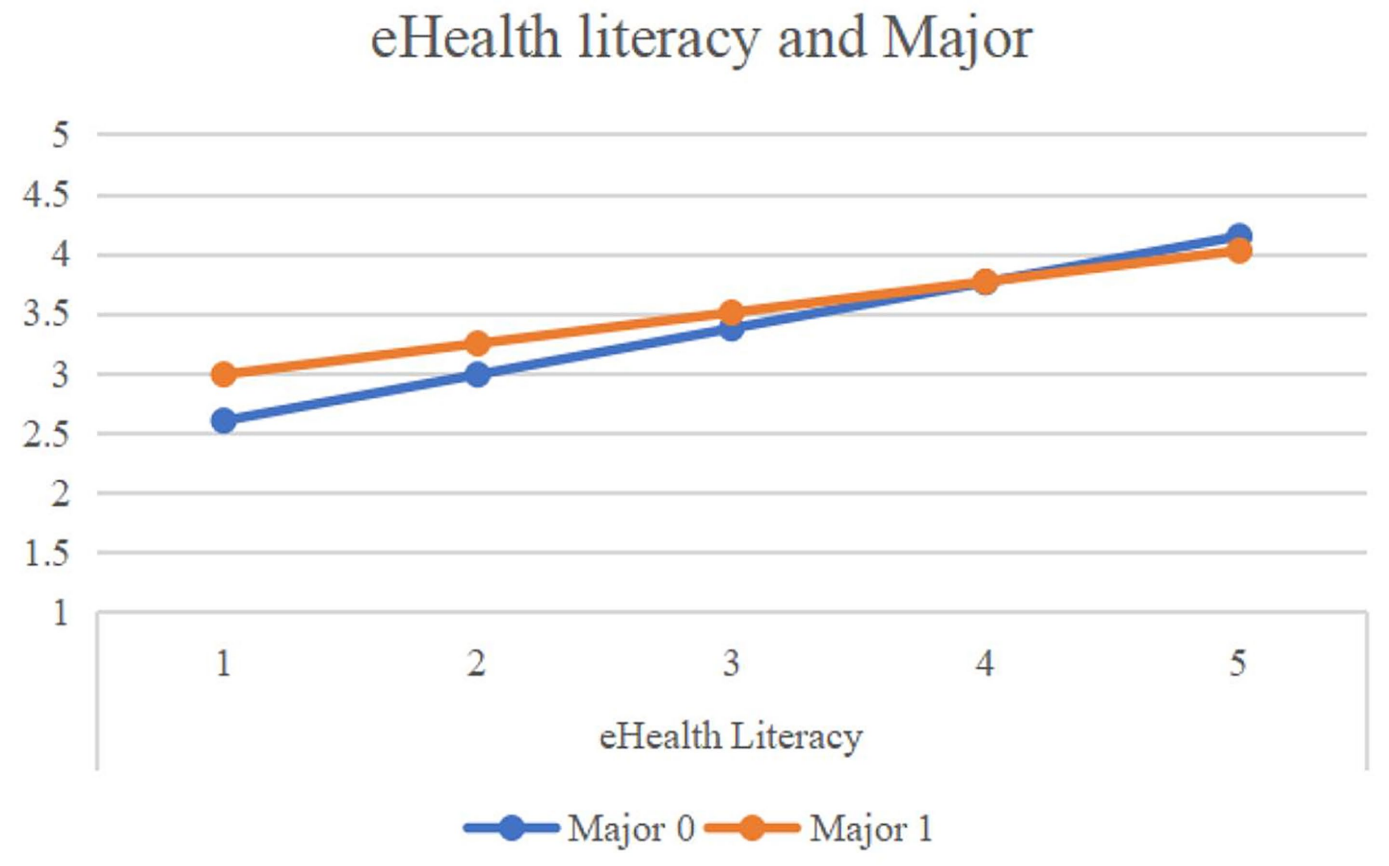
Moderating role of major in the relationship between eHealth literacy and healthy lifestyle.

In the relationship between eHealth literacy and healthy lifestyles (Table 7; Figure 4), eHealth literacy was a significant positive predictor of healthy lifestyles. The interaction term between eHealth literacy and major was statistically significant (*β* = −0.127, *p* < 0.001), indicating a significant moderating effect of major. Simple slope analysis revealed that while eHealth literacy positively predicted healthy lifestyles among both non-medical majors (β = 0.385, *p* < 0.001) and medical majors (β = 0.259, *p* < 0.001), this positive effect was significantly stronger in the non-medical student group.

**Table 7:** Moderating effect of major on the relationship between eHealth literacy and healthy lifestyles.

Variables	β	SE	t	95%CI
Main and interaction effects				
Constant	2.215	0.077	28.756***	2.064 ~ 3.970
eHealth literacy	0.386	0.015	26.273***	0.357 ~ 0.380
Major	0.514	0.134	3.842***	0.252 ~ 0.776
eHealth literacy × Major	−0.127	0.031	−4.089***	−0.188 ~ −0.066
Conditional effects (simple slopes)				
Major (non-medical)	0.386	0.015	26.273***	0.357 ~ 0.415
Major (medical)	0.259	0.027	9.487***	0.206 ~ 0.313

***p < 0.001.

**Figure 5:**
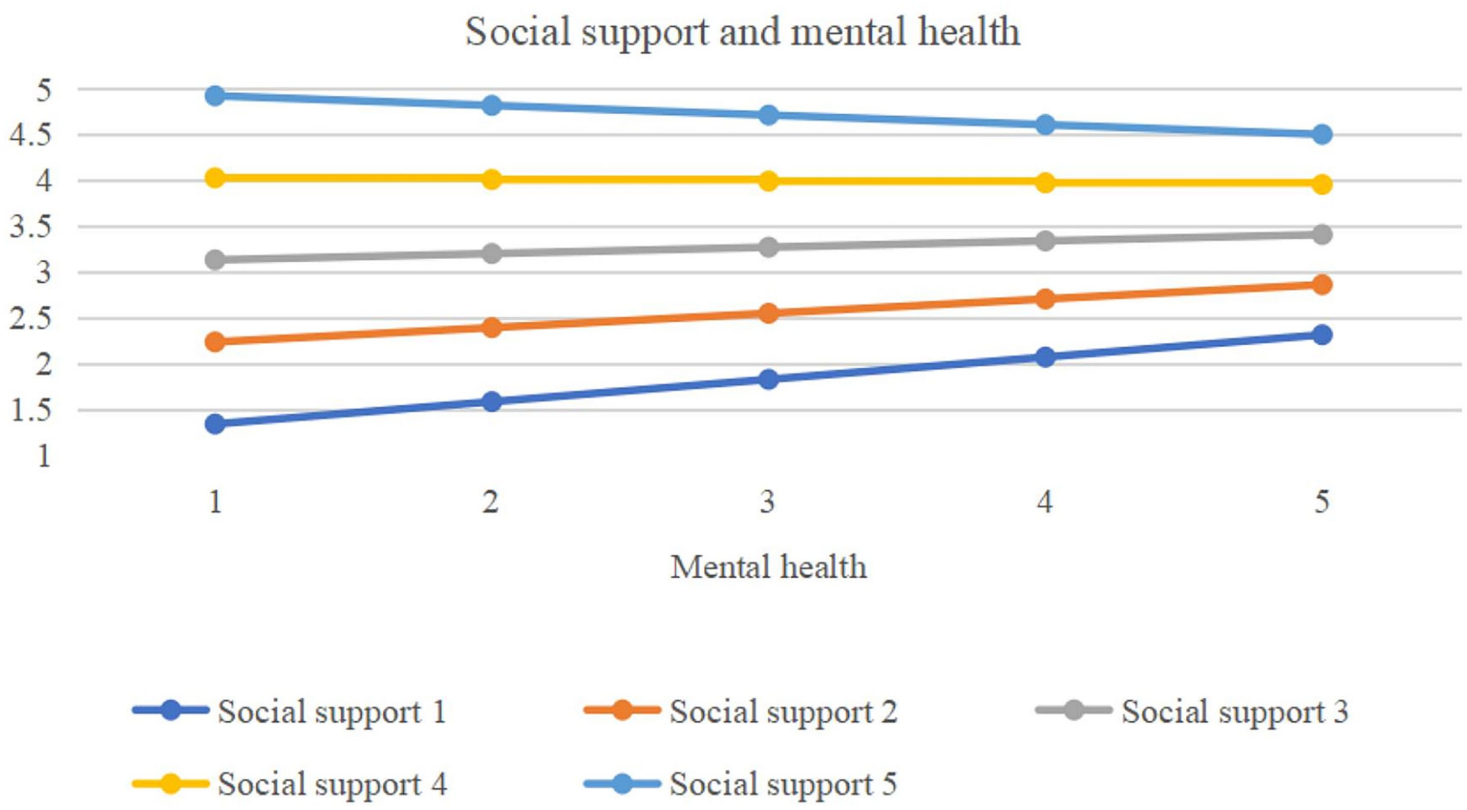
Moderating role of mental health in the relationship between social support and healthy lifestyle.

Social Support and Healthy Lifestyles (Table 8; Figure 5) demonstrated that social support was a significant positive predictor of healthy lifestyles The interaction term between social support and mental health was statistically significant (*β* = −0.087, *p* < 0.001). Simple slope analysis indicated that while social support positively predicted healthy lifestyles among both students with low (β = 0.763, *p* < 0.001) and high (β = 0.615, *p* < 0.001) levels of positive mental health, this effect was significantly stronger in the group with lower psychological well-being.

**Table 8:** Moderating effect of mental health on the relationship between social support and healthy lifestyles.

Variables	β	SE	t	95%CI
Main and interaction effects				
Constant	0.123	0.203	0.605	−0.276 ~ 0.522
Social support	0.980	0.046	21.094***	0.889 ~ 1.711
Mental health	0.329	0.061	5.368***	0.209 ~ 0.449
Social support × Mental health	−0.087	0.014	−6.188***	−0.114 ~ −0.059
Conditional effects (simple slopes)				
Low mental health	0.763	0.014	51.437***	0.734 ~ 0.792
High mental health	0.615	0.017	35.056***	0.581 ~ 0.650

*p < 0.01, **p < 0.001 indicate levels of statistical significance.

**Figure 6:**
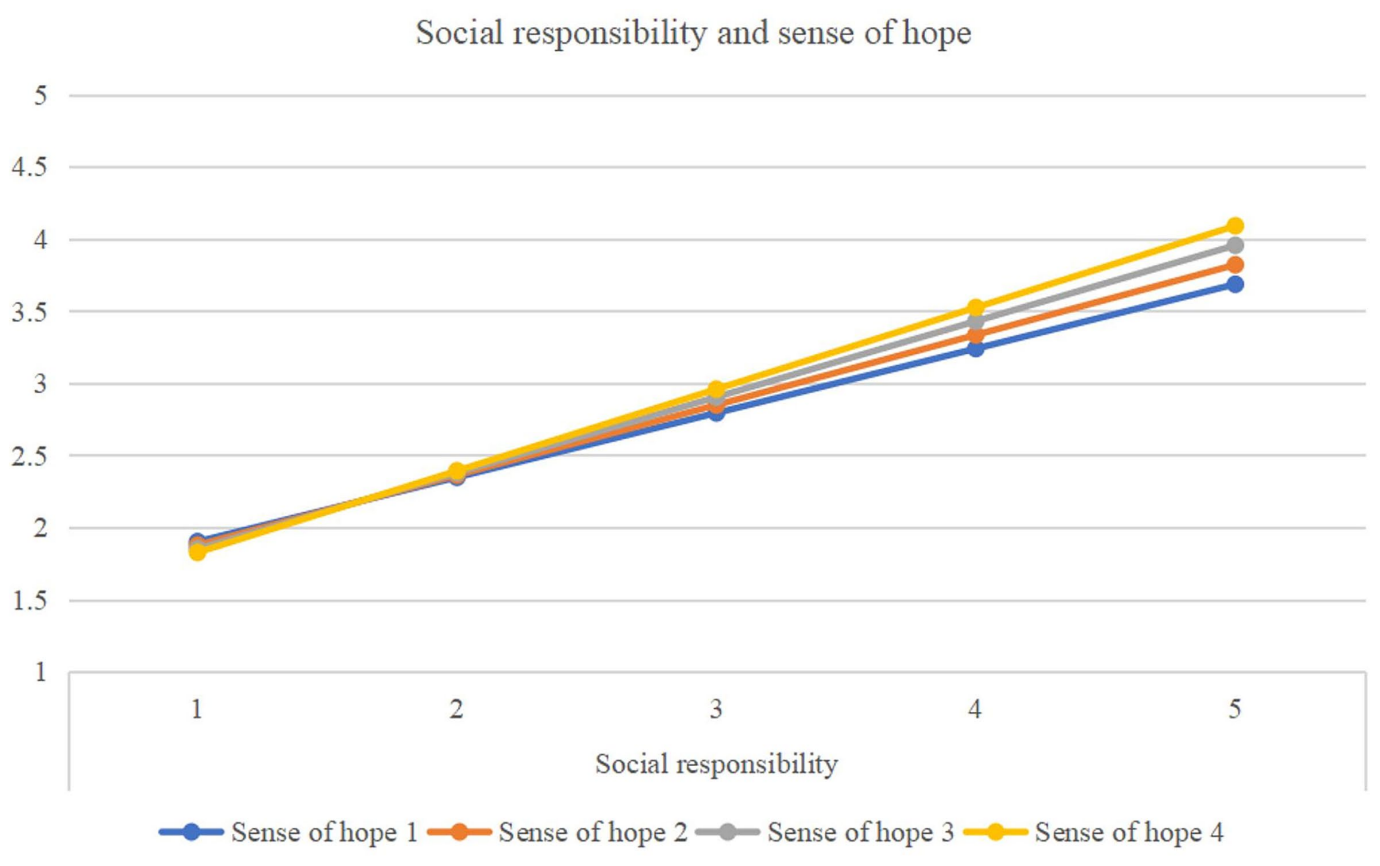
Moderating role of sense of hope in the relationship between social responsibility and healthy lifestyle.

Regarding the relationship between Social Responsibility and Sense of Hope on Healthy Lifestyles (Table 9; Figure 6), both variables emerged as significant positive predictors. A statistically significant interaction was found between social responsibility and sense of hope (β = 0.040, *p* < 0.001). Simple slope analysis revealed that the positive effect of social responsibility on healthy lifestyles was significantly stronger among students with a high sense of hope (β = 0.717, *p* < 0.001) compared to those with a low sense of hope (β = 0.602, *p* < 0.001).

**Table 9:** Moderating effect of sense of hope on the relationship between social responsibility and healthy lifestyles.

Variables	β	SE	t	95%CI
Main and interaction effects				
Constant	1.520	0.154	9.840***	1.217 ~ 1.823
Social responsibility	0.406	0.036	11.199***	0.335 ~ 0.477
Sense of hope	−0.065	0.025	−2.591**	−0.114 ~ 0.116
Social responsibility × sense of hope	0.040	0.006	6.995***	0.029 ~ 0.051
Conditional effects (simple slopes)				
Low sense of hope	0.602	0.013	47.707***	0.578 ~ 0.627
High sense of hope	0.717	0.015	49.189***	0.689 ~ 0.746

**p < 0.01, ***p < 0.001.

## 5 Discussion

### 5.1 Association between demographic characteristics and college students’ healthy lifestyles

This study employed a revised scale developed by Jiao et al. for measurement purposes (54). The scale encompasses multiple dimensions, systematically and comprehensively covering core domains of healthy living, thereby providing a holistic reflection of individuals’ overall healthy lifestyle levels. The average healthy lifestyles score among the 4,036 participants was 3.95 ± 0.74, indicating a moderately high level overall. Significant disparities were observed across different demographic characteristics. Specifically, female students, those in lower grades, single-child, and students from urban areas generally reported higher levels of healthy lifestyles. Female students scored significantly higher than male students, likely because they pay more attention to diet management and regular sleep schedules, consistent with previous research (65). Lower-grade students demonstrated significantly better healthy lifestyles than higher-grade students, a finding that contrasts with some previous studies (66). This observed difference may be attributed to the heightened academic and future-oriented pressures encountered by upper-grade students in Shandong Province. Renowned for its competitive educational environment, Shandong places a strong emphasis on academic achievement and post-graduation prospects. As college students progress to higher grades, they face escalating demands from major-specific coursework, internships, thesis writing, and career planning, which can diminish the time and energy available for health-promoting behaviors. Consequently, the decline in healthy lifestyles among upper-grade students appears to be shaped by the distinctive educational culture and structural pressures characteristic of this region. The single-child tend to have superior healthy lifestyles, likely because they often receive more concentrated resource investment and emotional concern within their families, fostering better health cognition and behavioral patterns (12). There are differences in the healthy lifestyles of college students with different gender, grade, and family type, so we should pay more attention to male students, upper-grade students, and non-only-child students.

### 5.2 The role of social responsibility as a core factor in association with healthy lifestyles

This study indicates that eHealth literacy, social support, and social responsibility all show a significant positive correlation with college students’ healthy lifestyles, among which social responsibility exhibits the strongest correlation, thus providing support for Hypothesis 1.

Under the framework of the COM-B model, eHealth literacy can be categorized as a capability factor. According to Guo et al., such capability involves the access, appraisal, and application of online health information (55). For college students, a higher level of eHealth literacy is reflected in their ability to effectively obtain health information from electronic media, to scientifically evaluate its credibility and applicability, and to a certain extent, effectively apply such information in addressing practical health problems (67). Under the framework of the COM-B model, social support belongs to the opportunity factor. According to Lou et al., it encompasses three dimensions: interpersonal networks, learning and living environments, and information environments (57). From our perspective, these elements create favorable conditions for college students to engage in health behaviors. Care and support from family members and peers are correlated with college students’ adherence to healthy behaviors (23, 24). A well-planned campus built environment is conducive to college students’ physical activity participation, while health information platform services are associated with students’ development of scientific health cognition, both of which are associated with the formation of healthy lifestyles (25, 26). Under the framework of the COM-B model, social responsibility is categorized as a motivation factor. Particularly within the Chinese sociocultural context, its connotation often extends beyond the scope of individual health management and is deeply integrated with traditional collectivist values. It encompasses individuals’ health responsibilities and public health responsibilities toward their families, communities, and even the nation. Zhu notes that increased awareness of social responsibility is associated with greater engagement in responsible practices, and our study also found that social responsibility is correlated with the adoption of healthy lifestyle behaviors (58). Unlike Western individualistic culture, the sense of social responsibility nurtured by collectivist education in China carries a deeper meaning: safeguarding one’s own health and that of others, as well as upholding collective interests. Based on current evidence, it is plausible that individuals with a strong sense of social responsibility may show a greater tendency to adopt preventive health behaviors and other relevant measures (31, 32).

Our study found that among the various factors linked to college students’ adoption of healthy lifestyle behaviors, social responsibility plays a relatively central and prominent role. From our perspective, its essence can be illuminated through Value Theory (27) and GITT. Social responsibility represents a final product of the entropy reduction process within an individual’s psychological world—a transition from “chaos and disorder” to “stability and order”—which is achieved by absorbing and integrating diverse external information through dynamic interactions (29). Interpretatively, this may suggest that eHealth literacy and social support’s regulation of information flow in digital and social environments correlates with the facilitation of this entropy reduction process. Specifically, eHealth literacy can serve as an accurate tool for information screening and integration, effectively identifying scientific health information and preventing cognitive confusion. On the other hand, social support establishes external resources via interpersonal networks, campus environments, and other channels, which appears to be conducive to accelerating the internalization of a sense of social responsibility. Collectively, these two factors are correlated with the reduction of psychological entropy, endowing social responsibility with a relatively stable and sustainable capacity that is associated with the facilitation of healthy behaviors, thereby positioning it as a core driver related to guiding college students to adopt healthy lifestyles.

### 5.3 Dual mediating role of social responsibility

The results showed that social responsibility played a mediating role between eHealth literacy, social support, and college students’ healthy lifestyles, forming two synergistic pathways and hypotheses 2a and 2b were supported. High eHealth literacy not only enriches students’ health knowledge but also strengthens their awareness of health obligations (68), enabling them to recognize the potential relevance of personal health to their families, schools, and society at large. Our study shows that eHealth literacy is associated with positive correlations on healthy lifestyle behaviors by enhancing students’ sense of social responsibility, which partially corroborates the pathway of “effectiveness of capability → motivation → behavior.” Social support not only provides direct material and emotional assistance (69) but also fosters a sense of belonging and collective identity (70). Our study also shows that social support is correlated with the facilitation of healthy lifestyle formation by stimulating students’ sense of social responsibility, verifying the pathway of “effectiveness of opportunity → motivation → behavior.”

### 5.4 Moderating effects

The results indicated that the positive correlation between eHealth literacy and college students’ healthy lifestyles varies by academic major, meaning this association is moderated by students’ academic disciplines. Specifically, the correlation between eHealth literacy and healthy lifestyles is more pronounced among non-medical students, and this finding provides empirical support for Hypotheses 3a and 3b. The underlying reason is that medical students, having received systematic curriculum training, typically possess a relatively comprehensive health knowledge system and practical skill set (71). Consequently, as a supplementary source of external information, eHealth literacy has a relatively limited incremental value for medical students, with its marginal utility showing a diminishing trend.

The study indicated that mental health level significantly moderates the relationship between social support and healthy lifestyles: the positive correlation between social support and healthy lifestyles shows a decreasing trend with the improvement of mental health level, which provides robust empirical support for Hypotheses 4a and 4b. College students with a high level of mental health exhibit strong emotion regulation ability, and their positive attitudes, psychological resilience, subjective well-being, and harmonious interpersonal relationships form a stable internal support system (59). Such students demonstrate a high level of autonomy in maintaining healthy behaviors and a low dependence on external support (33, 34). In contrast, those with a low level of mental health have vague health cognition and poor behavioral execution, and they are more reliant on external social support. When external resources are insufficient, they are more likely to engage in health risk behaviors (72).

The study demonstrated that as the level of hope increases, the positive correlation between social responsibility and healthy lifestyles becomes increasingly stronger, which partially validates Hypotheses 5a and 5b. Hope consists of two components: Dynamic thinking drives individuals to persistently pursue their goals, while pathway thinking involves the ability to formulate specific plans and strategies for achieving these goals (51). Individuals with high hope typically possess clear action pathways and strong intrinsic motivation, enabling them to internalize social responsibility as a motivation for healthy behaviors and amplify the positive correlation between social responsibility and healthy lifestyles (35). In contrast, those with low hope often lack clear health goals and confidence in achieving them (73). In such cases, social responsibility serves as an important external driver for maintaining healthy habits.

This study has several limitations. First, owing to human resource and geographical constraints, participants were primarily undergraduate students in Shandong Province, which may compromise the generalizability of the findings to some extent. Second, given the cross-sectional design adopted in this study, it is difficult to establish definitive causal relationships among the research variables. Third, as all data were collected via self-report at a single time point, common issues of methodological measurement bias may exert a certain association with on the research results.

## 6 Conclusion

Grounded in the COM-B theoretical model, this study investigates the formation mechanisms of healthy lifestyles among college students. It focuses on the interrelationships among the model’s three core components—capability, opportunity, and motivation—with particular emphasis on examining the role of social responsibility (as a motivational factor) within this framework. The findings reveal a dual role for social responsibility. On the one hand, as a motivational factor, it is positively associated with the adoption of healthy behaviors among college students. On the other hand, it acts as a critical mediator, bridging the relationships between eHealth literacy, social support, and healthy lifestyles. Specifically, eHealth literacy (a capability factor) and social support (an opportunity factor) are linked to the development of healthy lifestyles indirectly, through their associations with social responsibility as a core driver.

## 7 Implications

### 7.1 Practical implications

The research results provide the following targeted intervention paths for the construction of this system: Firstly, with motivation as the central focus, cultivating social responsibility should be positioned as the core strategy. Values-based education should guide students to internalize health behaviors as conscious actions benefiting both themselves and others. Secondly, when enhancing eHealth literacy, the moderating effect of academic discipline must be considered, with tailored foundational courses on health information discernment and application designed specifically for non-medical students. Furthermore, institutions should proactively develop and optimize campus social support networks while intentionally guiding students to gain positive experiences within these systems. This approach strengthens their sense of social responsibility and transforms supportive environments into sustainable drivers of health behaviors. Finally, the fundamental roles of sense of hope and mental health should be emphasized. Through positive psychology interventions and mental health services, students’ psychological capital can be enhanced, ensuring they possess the intrinsic resilience to maintain healthy behaviors when confronting challenges.

### 7.2 Theoretical implications

The core theoretical contribution of this study lies in its introduction of the Bayesian Mind sponge Framework and the Granular Interaction Thinking Theory, which significantly deepens and expands the theoretical connotation and explanatory power of the COM-B model. Specifically, the introduction of BMF [20]reveals the intrinsic mechanism of capability (eHealth literacy) from the perspective of information processing, elucidating how individuals filter, absorb, and integrate health information within complex online information environments based on their cognitive frameworks. The integration of Value Theory (27) and GITT (29), furthermore, explains the dynamic construction process of values from the perspective of motivation formation, emphasizing that through continuous interaction with multi-granular information in the environment, individuals constantly reassess and integrate their own values, ultimately forming stable, behavior-oriented motivations (social responsibility). This integrated framework connects the macro-structure of the COM-B model with micro-level cognitive-value mechanisms, providing a more comprehensive and dynamic analytical tool for systematically understanding and precisely intervening in complex health behaviors.

### 7.3 Policy implications

Based on the systematic findings of this study, universities and public health policy makers should shift from the current single-dimensional intervention model to a multi-level and systematic health promotion system construction.

### 7.4 Future research implications

Future research could be further developed in the following directions: First, longitudinal study designs could be adopted to capture the causal relationships in the formation process of healthy lifestyles. Second, in terms of research content, further exploration could be conducted on the moderating effects of other psychological traits within the COM-B model, while examining the generalizability of the integrated theoretical model proposed in this study to other populations. Third, regarding theoretical integration, attempts could be made to engage the integrated framework of this study in dialogue with more diverse theoretical perspectives, thereby further enriching our understanding of the complexity of healthy lifestyles.

## Data Availability

The raw data supporting the conclusions of this article will be made available by the authors, without undue reservation.
